# Quality of life and the related factors in spouses of veterans with chronic spinal cord injury

**DOI:** 10.1186/1477-7525-11-48

**Published:** 2013-03-18

**Authors:** Mohammad Hossein Ebrahimzadeh, Bibi-Soheila Shojaei, Farideh Golhasani-Keshtan, Seyed Hossein Soltani-Moghaddas, Asieh Sadat Fattahi, Seyed Mahdi Mazloumi

**Affiliations:** 1 Orthopedic and Trauma Research Center, Department of Orthopedic Surgery, University of Medical Sciences, Mashhad, Iran; 2 Endoscopic Research Center, Department of Surgery, University of Medical Sciences, Mashhad, Iran

**Keywords:** Quality of life, Caregiver, Iran-Iraq war, Spouses, Spinal cord injury, Veteran, Iran

## Abstract

**Background:**

The quality of life (QOL) of caregivers of individuals with chronic spinal cord injuries may be affected by several factors. Moreover, this issue is yet to be documented fully in the literature.

The purpose of this study was to evaluate the health related quality of life of spouses who act as primary caregivers of veterans with chronic spinal cord injuries in Iran.

**Methods:**

The study consisted of 72 wives of 72 veterans who were categorized as spinal cord injured patients based on the American Spinal Injury Association (ASIA) classification. Health related quality of life was assessed by the Short Form (SF-36) Health Survey. Pearson's correlation was carried out to find any correlation between demographic variables with SF-36 dimensions. To find the effect of the factors like age, employment status, duration of care giving, education, presence or absence of knee osteoarthritis, and mechanical back pain on different domains of the SF-36 health survey, Multivariate analysis of variance (MANOVA) was used.

**Results:**

The mean age of the participants was 44.7 years. According to the ASIA classification 88.9% and 11.1% of the veterans were paraplegic and tetraplegic respectively. Fifty percent of them had a complete injury (ASIA A) and 85% of the spouses were exclusive care givers. All of the SF-36 scores of the spouses were significantly lower than the normal population. Pearson's correlation demonstrated a negative significant correlation between both age and duration of caring with the PF domain. The number of children had a negative correlation with RE and VT.

**Conclusion:**

The burden of caregiving can impact the QOL of caregivers and cause health problems. These problems can cause limitations for caregiver spouses and it can lead to a decrease in the quality of given care.

## Background

The quality of life (QOL) of caregivers of spinal cord injured individuals is a complex issue that includes several factors such as physical, emotional, social and financial aspects [1]. There are studies that have claimed that other items such as care receiving characteristics, stress, coping strategies and social support can also impact quality of life [2].

The eight-year imposed Iran-Iraq war has caused various kinds of health issues based on different aspects not only for veterans, but also for their family caregivers who serve as these veterans main life support. Occurrence of spinal cord injury can cause physical and mental disabilities for the veterans who in turn can impact their family members and caregivers' quality of life [3,4]. Hence, the mental and physical health of veterans and their wives are mutually related to each other.

In Iran, similar to other countries where nursing of chronically injured patients is done by family members most of the time [5-7], wives are usually the ones who are most worried about their loved ones. These individuals, not only play a role as a wife, but also act as a caregiver with an increased work load and strain. In this situation, spouses have no choice but to make numerous kinds of adjustments within their mutual relationship. The effects of care giving for a patient with a chronic disease such as SCI have been documented in several studies [1,5,8-14]. In these individuals, social activities such as communication and relationships with friends and relatives are profoundly affected. In addition, most of them feel lonely and more frequently are depressed and fatigued, which ultimately results in lower health and QOL. Since pervious researchers have found a positive correlation between the quality of life of caregivers and the quality of provided care [11], we can imagine that a higher quality of life of caregivers can result in a higher satisfaction with life in patients that in turn can lessen the burden of caregivers as a feedback effect. In Iran, a study that examines the quality of life among spouses of SCI patients has not been well documented. So, the purpose of this investigation was to determine the quality of life and the related factors among caregiver spouses of veterans with long-term spinal cord injury.

## Methods

### Participants

A cross-sectional study was done on wives of veterans with a long term spinal cord injury (23-31 years having passed since being injury from the Iran-Iraq war), who live in Khorasan Razavi province in the northeast of Iran. Seventy-two wives (60%) out of a total of 120 wives who were invited to participate in this health survey participated in our study. This study was approved by Ethical Research Committee of Mashhad University of Medical Sciences.

After explaining the process of the study and our goals, the participants signed a consent form and started the face-to-face interview and filled out the questionnaires. Data was collected in paper printed forms and submitted to a computer SPSS data base.

### Design method

The study took around 6 months to be completed (from June 2011 until February 2012). This was a cross-sectional study. Demographic data of the caregiver spouses including age, level of education, employment status, duration of caretaking and/or living with the veteran, time of marriage (before or after injury) were asked and recorded in the forms (Table 1). In addition, the wives were examined for the presence of any mechanical lower back pain or knee osteoarthritis and suspected cases were given an X-ray to confirm diagnosis. Meanwhile, the neurological status of their husbands was documented based on the American Spinal Injury Association (ASIA) guidelines.

**Table 1:** **Characteristics of the seventy**-**two caregiver spouses and their seventy**-**two husbands with chronic spinal cord injury**

***Characteristics***	**N (%)**		**MCS**		**MANOVA**		**PCS**		**MANOVA**	
			**Mean (SD)**		***p***		**Mean (SD)**		***p***	
	***Spouses (care giving)***	***Patient of spinal cord injury***	***Spouses (care giving)***	***Patient of spinal cord injury***	***Spouses (care giving)***	***Patient of spinal cord injury***	***Spouses (care giving)***	***Patient of spinal cord injury***	***Spouses (care giving)***	***Patient of spinal cord injury***
*Age*										
≥44 years	44(61.1)	17(23.5)	40.9(13.3)	54.1(13.6)	0.92	>0.001	40.2(10.6)	34.7(26.7)	0.57	>0.001
< 44 years	28(38.9)	55(76.5)	40.7(12.5)	48.9(11.3)			41.3(10.5)	30.9(8.54)		
*Employment status*										
Unemployed	64(88.9)	56(76.9)	38.4(13.2)	49.7(11.7)	0.08	>0.001	40.9(11.9)	31.6(15.1)	0.11	>0.001
Employed	8(11.1)	16(23.1)	47.9(15.6)	51.3(12.9)			48.5(11.5)	32.2(9.96)		
*Duration of care*-*giving*										
≥22 years	24(33.3)	-----	39.8(13.3)	-----	0.63	-----	40.7(12.5)	-----	0.33	-----
<22 years	48(66.7)	-----	38.1(14.6)	-----			43.7(10.8)	-----		
*Knee osteoarthritis*										
No	32(44.4)	------	41.1(14.0)	------	0.30	-----	45.3(11.6)	-----	0.015^*^	-----
Yes	40(55.6)	-----	37.8(13.3)	-----			38.5(11.5)			
*Mechanical back pain*										
No	40(55.6)	-----	41.2 (14)	-----	0.19	-----	45.3(11.6)	----- -----	0.004^**^	-----
Yes	32(44.4)	-----	37.09(13.1)	-----			37.2(11.8)			
*Education*										
High school or less	71(98.6)	51(71.1)	38.1(13.0)	49.8(12.3)	0.22	>0.0001	40.5(12.3)	32.5(14.9)	0.21	>0.0001
University degree	1(1.4)	21(28.9)	42.2(15.3)	51.08(10.4)			44.9(10.9)	28.9(10.7)		
*Number of children*										
>= 2	37(51.4)	-----	37.4(14.3)	-----	0.3	------	41.1(12.7)	-----	0.8	------
< 2	35(48.6)	------	40.5(12.8)	------			40.7(11.2)	------		

^*^Statistically significant at the 0.05 level.

^**^Statistically significant at the 0.01 level.

### Questionnaire

#### SF-36

The SF-36 is an instrument that has been used for a long time to assess health related quality of life among individuals with spinal cord injury. This survey has 36 items and measures eight domains: Physical Functioning (PF), Social Functioning (SF), Role-Physical (RP), Bodily Pain (BP), Mental Health (MH), Role-Emotional (RE), Vitality (VT), and General Health (GH). Higher scores in each of the 8 subscales indicate better functioning and well-being. Validity and reliability of the SF-36 survey has been approved in the Persian language [15]. For each section, a person with a higher score will have a better function (0-100).

### Statistical analysis

The t-test was applied to compare between the SF-36 values of the spouses and the normal female population in Iran (2165 individuals). Apart from this, Pearson's correlation coefficient (r) was carried out to find the relationship between demographic variables like age, education, and duration of living with the veteran and the SF-36 subscales. Again, to find the effect of the factors such as age, employment status, duration of the caretaking, education, presence or absence of knee osteoarthritis, and mechanical back pain with the main scores of the quality of life (PCS and MCS), multivariate analysis of variance (MANOVA) was used. SPSS 16 was used to carry out the needed analysis. P value of less than 0.05 was considered to be satisfactory.

## Results

All of the 72 participants were caregiver spouses of veterans with chronic spinal cord injuries. Eight individuals (11%) were employed at the time of the interview and 64 wives (89%) were unemployed and thus worked full time at home. The mean age of the participants was 44.7 years with a minimum and a maximum of 31 to 66 years respectively (SD = 6.55). The range of the number of their children was from 0 to 6 (mean = 1.9, SD =1.55). Based on ASIA categorization, 88.9% of the veterans (64 individuals) had a paraplegic lesion and 11.1% of them (8 individuals) were tetraplegia. Apart from this, 36 veterans (50%) had a complete SCI (ASIA A) and the remaining were half diagnosed with an incomplete lesion (ASIA B or C or D). Demographic data is summarized in Table 1.

The results showed that there was a negative significant correlation between the ages of caregivers with the PF domain (p=0.004, r=-0.034) of the SF-36 health survey. Also, there was a significant correlation between the education level with the PF domain (p=0.041, r=0.241), VT (p=0.026, r=0.263) of the SF-36 survey.

The number of children had a negative correlation with VT (p=0.021, r=-0.282) and RE (p=0.049, r=-0.242) domains of the SF-36 survey.

**Figure 1:**
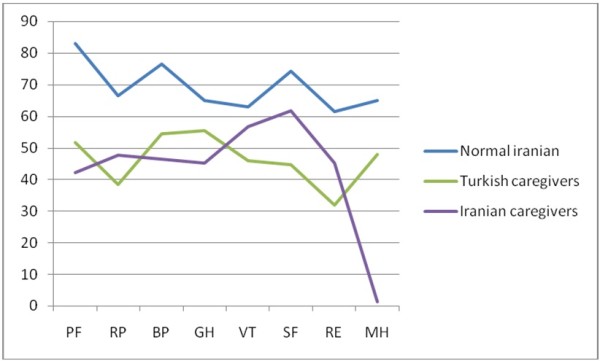
**Comparison of the SF**-**36 scores between veterans' spouses of this study to a Turkish study (50 persons), and the normal Iranian population (2165 females).**

Figure 1 and Table 2 demonstrate the results of the t-test done to compare the SF-36 values between Iranian spinal cord veterans` wives (present study), the normal population of Iranian women [1], and Turkish spinal cord caregivers [2]. All sub-scales of the SF-36 health survey of the Iranian spinal cord caregiver spouses were lower than the normal Iranian women with a significantly high difference (p<0.01).

**Table 2:** **Compare the SF**-**36 values between wives** (**present study**), **the normal population of Iranian women**[1], **and Turkish caregivers**[2]

**Column 1**	**Normal Iranian**	**Turkish caregivers**	**Iranian caregivers**
PF	82.9	51.7	42.05
RP	66.5	38.5	47.5
BP	76.4	54.3	46.3
GH	65	55.4	45
VT	62.9	45.86	56.6
SF	74.2	44.72	61.6
RE	61.4	32	45.1
MH	65	48	53.8

However, the test yielded a different result in comparing the SF-36 scores of Iranian and Turkish caregivers [12]. The Iranian spouses had a better function score in almost all domains except VT and GH compared to Turkish caregivers. Furthermore, significant differences occurred for PF and SF domains (*p*<0.05). Figure 1 includes more details regarding this comparison.

On the other hand, the Pearson's correlation demonstrated a negative significant correlation between both the age of the caregivers and duration of living with the veteran in the PF domain (p=0.014, r=-0.29) of the SF-36 health survey. Apart from this, a positive significant correlation was observed among educational status and PF as well as in the VT domain. Also, the number of children had a negative significant correlation with RE and VT (Table 3).

**Table 3:** **Pearson**'**s correlation test between the demographic values and the SF**-**36 variables**

***SF***-***36***										
**Items**	**PF**	**RP**	**BP**	**GH**	**VT**	**SF**	**RE**	**MH**	**PCS**	**MCS**
*Age*										
r	-.340^*^	.122	-.084	.067	.014	-.084	-.187	-.016	-.042	-.059
p	.004	.308	.482	.577	.904	.484	.115	.897	.726	.625
*Education*										
r	.241^*^	.019	.067	.063	.263^*^	.022	.106	.187	.072	.138
p	.041	.873	.577	.600	.026	.856	.377	.115	.546	.249
*Number of children*										
r	-.186	-.012	-.072	-.009	-.282^*^	-.089	-.242^*^	-.034	-.044	-.168
p	.132	.922	.563	.941	.021	.473	.049	.786	.726	.174
*Duration of care*-*giving*										
r	-.290^*^	.062	-.154	.015	-.037	-.066	-.164	-.096	-.075	-.092
p	.014	.605	.197	.899	.755	.579	.169	.422	.534	.443

^*^Correlation is significant at the 0.05 level (2-tailed).

## Discussion

Today, research on quality of life and health status are quantified by standard instruments in difficult domains of human life. It is assumed that caring for people with chronic spinal cord injury may affect the primary caregivers` health and this point should be greatly considered as long as the injury exists and the caregiver provides care [16,17].

In agreement with the finding of Rahimi-Movaghar et al. who found a high burden for SCI, we assumed a lower quality of life among the caregivers of SCI individuals due to the high work load. The results of our study confirmed this hypothesis by demonstrating lower SF-36 scores among caregiver wives comparing to normal Iranian women. This finding also has been confirmed by other researchers [1,9,12,18,19].

Naturally, when the people become older they will experience a decline in their physical function [8]. Likewise, we found that with the increase of the caregivers’ age there was a reduction in the physical component (PF) of the quality of life [10]. However, when the level of education of the participants increased, it resulted a rise in the level of the quality of life (better PF and VT). We had also expected that a higher education and better knowledge of different life circumstances would bring about a better life situation for them. Conversely, other factors such as having more children, being employed, and a longer duration of caregiving, all of which intensifies the caregiver’s work load and burden, can be a contributing factor of lower physical function and quality of life.

Another influencing factor which can negatively affect the physical function is the duration of caregiving [10]. The duty of caring for a patient with a chronic disease can increase one’s levels of fatigue and result in a lower QOL [13]. A possible explanation for this is that the pressure of the burden can impact the physical health of the caregivers and cause problems like mechanical back pain and knee osteoarthritis as well as having a lower general health condition. These problems can cause limitations for the caregivers and we can anticipate a decline in the quality of the care given by them. Shimoyama et al. after assessing patients and their caregivers discovered that a lower level of physical ability in caregivers can be due to various factors including being less healthy due to physical and mental issues [8]. According to this, we can infer that a lower level of physical function in the participants of our survey can be the result of physical problems such as chronic back pain or knee osteoarthritis. Apart from these, the emotional problems which they have encountered during the period of caregiving can be another contributing factor.

Considering that most of the time spouses play the roles of the caregivers [5-7] we can infer that chronic diseases like Alzheimer or spinal cord injury can cause a heavy burden for caregiver spouses and in order for the caregiver to continue to provide care a strong emotional tie with the SCI patient is needed. In our study we observed this, as caregivers expressed their powerful connection to their patients to be the main reason of continuing their duty. Moreover, they believed that their religious beliefs have had a positive impact in their thinking which leads them to be optimistic.

As psychological issues play an important role in such a situation [4], investigations have been done to evaluate the frequency of depression and anxiety and their effects on caregivers. Chan et al. [14] as well as Hadrys et al. [10] investigated the frequency of psychological problems in the caregivers of patients with SCI and mental disorders respectively. [1-3] They reported that caregiving can have a negative effect on the mental health of these persons and claim that depression and anxiety are highly prevalent among them. Correspondingly, we discovered a lower rank in the wives' mental components (VT, SF, RE, MH) of the SF-36 health survey. Although this finding can be representative of lower mood function in the spouses of veterans with chronic spinal cord injury, their psychological health can be evaluated by more specific instruments like Hospital Anxiety and Depression Scale (HADS) or Patient Health Questionnaire (PHQ-9) for a more precise result.

From the results of the study we can conclude that determinants of negative effects on the quality of life of the caregivers are higher age, a higher number of children, and long duration of caregiving, and having diseases like knee osteoarthritis or mechanical back pain. Also, by conducting a more detailed study regarding their psychological health and burden of caregiving by using a specific questionnaire such as the Zarit Burden Interview [20], which has been shown to be a competent instrument in different countries, we will be able to quantify the amount of burden in caregivers of individuals with chronic diseases like SCI. Furthermore, comparison of the quality of life between caregivers and patients will enable us to explore the effect of different factors bilaterally. In this study, one of our limitations was not being able to evaluate the problems which both encounter outside their residential place, which will give us valuable information regarding environmental issues in order to find better strategies and remove any possible obstacles.

To sum up, various items can influence the QOL among the caregivers or spouses of patients with a chronic disease like spinal cord injury. The chronicity of the problem can negatively affect the life of these caregivers as well as their QOL. However, with better support and more education, and generally by modifying the contributing factors, we can anticipate a better quality of life for the caregiver spouses and as a result improved care of spinal cord injured veterans. As it has been suggested by Devins et al and Hadrys et al that by instructing caregivers and organizing patient education programs and family interventions, quality of life can be improved for both caregiver spouses and their disabled husbands [10,21]. Therefore, we can facilitate the management of a disabling condition such as SCI and improve the psychosocial life for both the caregivers and their patients.

## Competing interest

The authors declare that they have no competing interests.

## Authors’ contributions

MHE participated in the design of the research, implementation of the study protocol and interpretation of data and publication of the manuscript. BSSH visited the spouses of spinal cord injured patients as well. FG participated in the analysis and interpretation of data and drafting of the manuscript. SHS and MM contributed to the drafting of the manuscript. All of the authors read and approved the final manuscript.
